# Prediction of recurrence using hematologic and urinary markers in intravesical Bacillus calmette Guerin treated bladder cancer

**DOI:** 10.1038/s41598-025-14974-1

**Published:** 2025-10-10

**Authors:** Mecit Celik, Muhammed Emin Polat, Mustafa Karaaslan, Öner Odabas, Fatma Meric Yilmaz

**Affiliations:** 1 Department of Urology, Ergani State Hospital, Adnan Menderes, Ergani, 21950 Diyarbakir, Turkey; 2 Department of Urology, Ankara Bilkent City Hospital, Ankara, Turkey; 3 Department of Urology, Bayindir Kavaklidere Hospital, Ankara, Turkey; 4 Faculty of Medicine, Yildirim Beyazit University, Ankara, Turkey

**Keywords:** Bacillus Calmette-Guérin, Flow cytometry, Immunology, Non-Muscle invasive bladder cancer, Recurrence, Tumour biomarkers, Tumour immunology, Urological cancer

## Abstract

Intravesical bacillus Calmette-Guérin (BCG) therapy reduces recurrence in non-muscle invasive bladder cancer (NMIBC). Various scoring models have been developed to predict recurrence, and this study aims to improve them by analyzing hematologic parameters and urinary immune cells. This study involved 88 bladder cancer patients treated with BCG between January 2022 and January 2023. Preoperative hematological parameters were recorded from two separate blood samples. Urine samples were collected before and after the first and sixth BCG instillations and analyzed by flow cytometry to determine the proportions of T cells, neutrophils, and myeloid-derived suppressor cells (MDSCs). Results were compared based on recurrence status and within-group changes. Six patients died from non-cancer causes, and five were excluded due to irregular follow-ups or external monitoring. Of the remaining 77 patients, followed for an average of 30 months, 12 experienced recurrence, and 65 did not. No significant differences were found in clinical or histopathological factors. Urine analysis revealed a higher Neutrophil/T cell ratio in patients with recurrence, while the T/MDSCs ratio increased significantly in the non-recurrent group post-BCG. Additionally, the MDSCs/Total Cell ratio significantly decreased post-treatment in the non-recurrent group. Peripheral blood analysis showed no significant differences in Neutrophil-Lymphocyte Ratio (NLR) and Lymphocyte-Monocyte Ratio (LMR). The results suggest a T cell-dominant immune response may prevent recurrence, while an MDSCs-dominant environment increases the risk. Changes in urinary T/MDSCs, MDSCs/Total Cell, and Neutrophil/T cell ratios may serve as potential biomarkers for predicting recurrence risk shortly after induction therapy.

## Introduction

Bladder cancer (BC) is the seventh most common malignancy among men worldwide^1^with approximately 75% of patients diagnosed with NMIBC at presentation^2^.

The standard treatment includes transurethral resection of the bladder tumor (TURBT), often followed by intravesical chemotherapy or BCG immunotherapy^3^. Despite these strategies, recurrence and progression remain significant challenges. According to the European Organization for Research and Treatment of Cancer (EORTC), the 1-year and 5-year recurrence rates are 25.9% and 41.3%, respectively^4^. Although current risk stratification relies on clinical and histopathological data, emerging biomarkers are being investigated to better predict outcomes^5^.

Early detection of recurrence and progression is critical in BC follow-up^6^prompting research into the role of inflammation and tumor immunology^6,7^. Inflammatory markers such as NLR have been widely studied^7^and T-cell responses to BCG therapy have been shown to play a key role in anti-tumor immunity, primarily within the tumor microenvironment (TME)^8^.

Among the immunosuppressive components of the TME, MDSCs are notable for their ability to inhibit T-cell activity and promote tumor progression^8^. As a non-invasive alternative to tissue-based analysis, urine-based flow cytometry has emerged as a promising tool for assessing the immunological landscape^9^.

In this study, we aimed to evaluate urinary T cells, Neutrophil and MDSCs, along with peripheral blood markers such as NLR and LMR, as potential predictors of recurrence in NMIBC patients undergoing BCG therapy.

More accurate prediction of recurrence could help optimize the administration of intravesical BCG therapy, which is associated with toxicity, sepsis-related mortality, and challenges in production and supply, ensuring that this costly treatment is allocated to appropriate patients^10,11^. In patients experiencing recurrence during BCG immunotherapy, additional BCG instillations have been linked to an increased risk of progression^12^. Given that muscle invasion developing during follow-up carries a worse prognosis than muscle invasion detected at initial diagnosis, better prediction of recurrence could prevent delays caused by BCG therapy and facilitate earlier consideration of radical surgical interventions as a viable option^12,13^.

Another objective of this study is to identify the key immune cell populations contributing to therapeutic resistance in the recurrence group. Understanding these mechanisms may pave the way for the development of targeted therapeutic strategies against these resistant cell populations^14^.

## Materials and methods

### Ethical approval

Ethical approval was obtained from the Ethics Committee of Clinical Research No. 2 at Ankara Bilkent City Hospital (Approval No: E2-20-95, 30/12/2020). The study began two years later due to global BCG shortages and COVID-19-related delays. Research on participants was conducted in accordance with the Declaration of Helsinki.

### Patient selection and study design

Between January 2022 and January 2023, 88 patients who underwent TURBT at Ankara Bilkent City Hospital following a preliminary diagnosis of bladder tumor were included. Informed consent was obtained from all patients included in the study. All patients had clinically and pathologically confirmed high-risk disease and received BCG therapy. Sample size was estimated using G*Power (α = 0.05, power = 0.90, effect size = 0.7). Exclusion criteria included prior bladder cancer, non-urothelial subtypes, systemic inflammatory diseases, contraindications to BCG (e.g., immunosuppression, pregnancy, BCG allergy), incomplete induction (fewer than six doses), irregular follow-up, or refusal to participate.

### Flow cytometric analysis

**Fig. 1:**
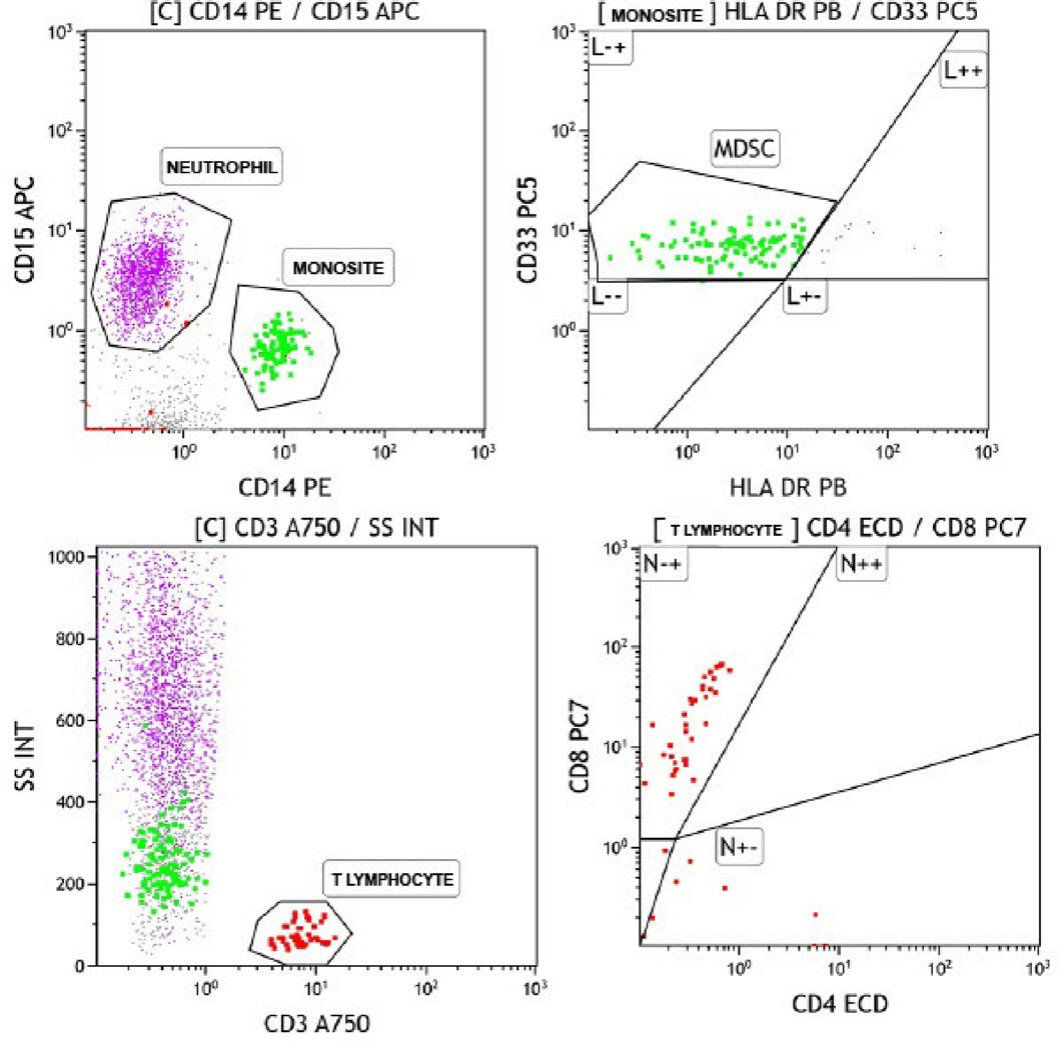
Dot plot graph of flow cytometry analysis performed on a representative patient.

Four urine samples per patient were collected in sterile containers and analyzed the same day at the Flow Cytometry Laboratory of Ankara Bilkent City Hospital using a 10-color Navios flow cytometer (Beckman Coulter). Due to BCG-induced local immune activation, PMNLs were detectable in urine. Samples were processed per manufacturer’s protocol: centrifuged at 4,000 rpm for 20 min, and 100 µL of the pellet was incubated with fluorochrome-conjugated antibodies (CD45-KO, CD33-PC5, CD14-PE, CD3-A750, CD8-PC7, CD4-ECD, CD15-APC, HLA-DR-PB) for 15 min in the dark. After washing and resuspension in IsoFlow, samples were analyzed via Kaluza software. The proportions of neutrophils, monocytes (notably MDSCs), and T cells were reported as dot plots (Fig. 1). M-MDSC cells were defined as CD14 HLA-DR^−^/low cells. Neutrophils (CD15^+^, CD14^−^) and monocytes (CD15^−^, CD14^+^) were identified based on CD15/CD14 dot plots. Monocytes were further gated, and M-MDSCs were defined as CD33^+^ HLA-DR^−^/low cells using a CD33/HLA-DR dot plot. CD3^+^ cells were gated to ide^+^ntify T cells, and subsequent analyses were performed to characterize CD4^+^ and CD8^+^ T cell subsets.

### Cell ratio calculations

To investigate the immunological dynamics in the tumor microenvironment, we calculated various cell population ratios based on flow cytometry data. Ratios such as T cell/MDSC, and MDSC/ Total cells were used to explore the balance between immune-suppressive and immune-effector cells. These combinations were intentionally selected to highlight different biological relationships and reflect shifts in immune dominance. No single cell type was used as a fixed reference; instead, diverse ratios allowed us to evaluate the relative abundance and potential functional significance of each population in the context of BCG response and recurrence.

### Statistical analysis

Statistical analyses were conducted using the Statistical Package for Social Sciences (SPSS), version 22.0 (SPSS Inc., Chicago, USA). The Kolmogorov-Smirnov test was used to assess the normality of data distribution. In the descriptive statistics section, non-parametric data were reported as mean ± standard deviation (SD). Based on normality test results, Mann-Whitney U tests were used for comparisons between groups. The Wilcoxon test was applied to assess within-group changes before and after BCG therapy. A p-value of < 0.05 was considered statistically significant for clinical variables.

## Results

 A total of 88 patients were included in the study. Among them, six patients died during the follow-up period, and five patients were lost to follow-up or continued their treatment at other centers. Demographic, clinical, and histopathological data of the 77 patients included in the final analysis are presented in Table 1.

 No significant differences were observed between the recurrence and non-recurrence groups in terms of gender, age, number of tumors, tumor size, smoking status, presence of concomitant CIS. (Table 1).

**Table 1:** Demographic, clinical and histopathological data of patients.

	Recurrence negative *n*:65(%)	Recurrence positive *n*:12(%)	*p*
Sex, F/M	7/58	3/9	0.18*
Primary pathology			0.56*
TaLG	7 (10.8%)	2 (16.7%)	
TaHG	17 (26.2%)	2 (16.7%)	
T1LG	6 (9.2%)	0 (0%)	
T1HG	35 (53.8%)	8 (66.7%)	
Concomitant CIS	10 (15.4%)	2 (16.7%)	0.91*
Smoke			0.17*
Non-smoker	11 (17.5%)	5 (41.7%)	
Ex-smoker	39 (61.9%)	5 (41.7%)	
Smoker	13 (20.6%)	2 (16.7%)	
Age, year	66.085 ± 9.783	68.417 ± 6.908	0.5 †
Number of Tumors	1.848 ± 1.064	1.5 ± 0.798	0.29 †
Tumor size(cm)	4.229 ± 1.901	4.792 ± 1.499	0.12 †
Follow-up period, (months)	30.966 ± 5.179	28.5 ± 3.261	0.17 †

* Chi-Square test † Mann Whitney U test.

The mean follow-up period was 30 months, during which a total of 12 patients experienced recurrence. Pathological examination revealed that five of these patients had T2HG disease. Among these, four cases were identified through surveillance cystoscopy, while one case was diagnosed following radical cystectomy. Additionally, surveillance cystoscopy detected three cases of T1HG, three cases of T1LG, and one case of TaHG. (Table 2).

**Table 2:** Primer and recurrence pathology in recurrence-positive patients.

	First pathology	Recurrence pathology
TaLG	2(16.7)	0(0)
TaHG	2(16.7)	1(8.3)
T1LG	0(0)	3(25)
T1HG	8(66.7)	3(25)
T2HG	0(0)	5(41.7)

A comparative analysis of NLR and LMR obtained from two complete blood counts performed at the time of initial NMIBC diagnosis showed no statistically significant differences between the recurrence and non-recurrence groups (Table 3).

Flow cytometric analysis was performed on four urine samples collected from each patient, and the comparison of neutrophil-to-T-cell ratios between the recurrence and non-recurrence groups revealed statistically significant differences at pre-BCG, pre-6th BCG, and post-6th BCG time points (*p* = 0.048 *p* = 0.005 and *p* = 0.02 respectively). However, although the neutrophil-to-T-cell ratio was higher in the recurrence group after the first BCG instillation, the difference did not reach statistical significance. (Table 3).

**Table 3:** Comparative analysis of outcomes of patient groups according to recurrence status.

	Recurrence negative (*n*:65)	Recurrence positive(*n*:12)	P*
Neutrophil /T Cell_1B	236.08 ± 375.31	321.81 ± 306.37	**0.048**
Neutrophil /T Cell_1A	128.03 ± 143.02	198.68 ± 197.29	0.08
Neutrophil /T Cell_6B	63.35 ± 153.58	107.64 ± 85.54	**0.005**
Neutrophil /T Cells_6A	54.14 ± 71.832	69.452 ± 53.430	**0.023**
LMR	4.725 ± 1.607	4.125 ± 1.623	0.095
NLR	2.527 ± 1.009	2.798 ± 0.99	0.247

1B: Pre-1st BCG urine sample analysis 1 A: Post-1st BCG urine sample analysis 6B: Pre-6th BCG urine sample analysis 6 A: Post-6st BCG urine sample analysis LMR: Lymphocyte to Monocyte Ratio NLR: Neutrophil-to-Lymphocyte Ratio *Mann-Whitney U test.

**Table 4:** Comparison of urine sample analyses within groups between pre-first BCG and post-final BCG instillation.

	Recurrence negative(*n*:65) p*		Recurrence positive (*n*:12) p*	
MDSCs/Total cell_1B	1.309 ± 1.209	**0.003**	0.779 ± 0.65	0.58
MDSCs/Total cell _6A	0.771 ± 0.543		0.810 ± 0.421	
Tcells/MDSCs_1B	1.267 ± 1.598	**< 0.001**	1.058 ± 0.793	0.07
Tcells/MDSCs_6A	4.68 ± 4.614		1.914 ± 0.867	

1B: Pre-1st BCG urine sample analysis, 6 A: Post-6st BCG urine sample analysis *Wilcoxon test.

**Fig. 2:**
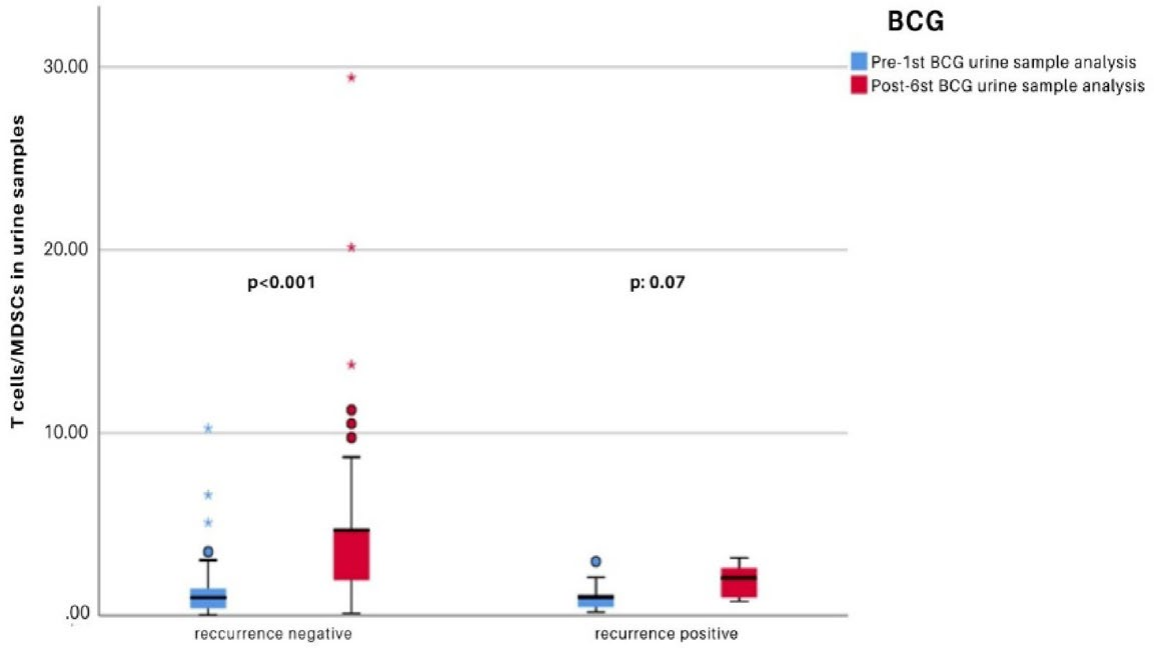
Comparison of Changes in the Tcells/MDSCs ratio in urine samples collected before the first treatment and after the final treatment among patient groups.

A separate analysis was conducted within each group to compare the flow cytometric findings of urine samples obtained before BCG therapy and after the final induction dose. As shown in Fig. 2, in the non-recurrence group, the Tcells-to-MDSCs ratio significantly increased following the 6th BCG instillation compared to pre-treatment levels.( (*p* < 0.001) However, although the Tcells-to-MDSCs ratio also increased in the recurrence group, this change was not statistically significant ( *p* = 0.07) (Fig. 2; Table 4).

**Fig. 3:**
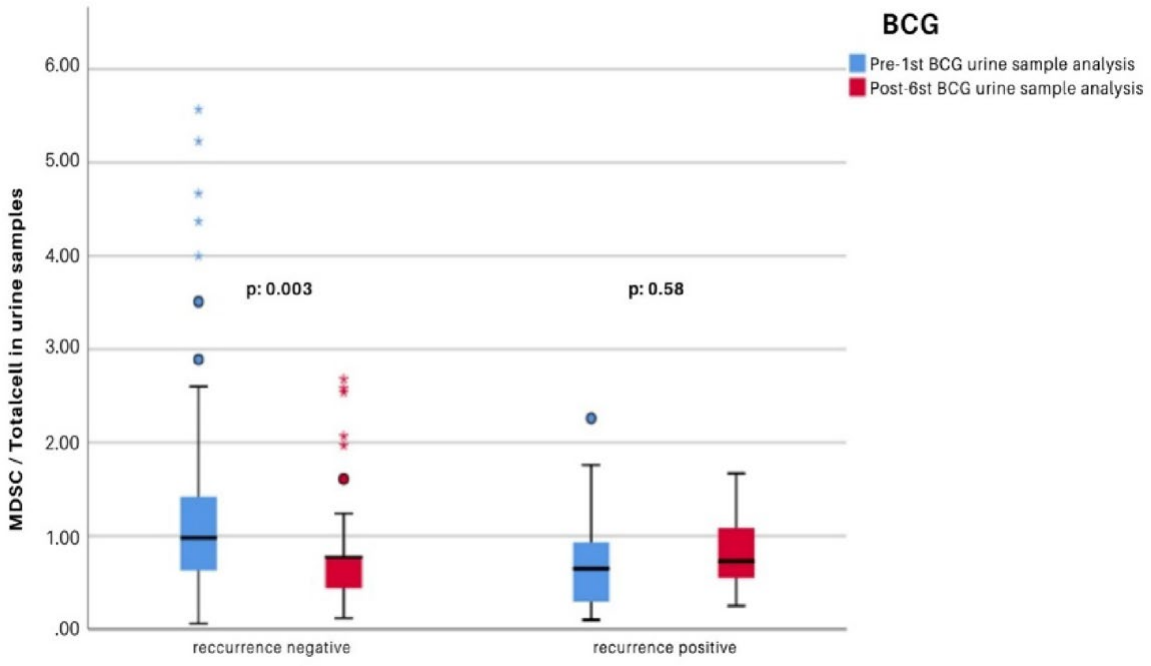
Comparison of changes in the MDSCs/Total cell ratio in urine samples collected before the first treatment and after the final treatment among patient groups.

 During BCG immunotherapy, a gradual increase was observed in the absolute counts of all immune cell subsets tested in the urine. However, the proportion of MDSCs among total urinary cells significantly decreased in the non-recurrence group, whereas it did not decrease in the recurrence group and instead showed an increasing trend (*p* = 0.003) (Fig. 3).

## Discussion

 NMIBC has unpredictable recurrence and progression patterns, and existing models like the EORTC risk scores rely mainly on clinical and pathological data, often failing to predict outcomes accurately^4,15–17^. These models may underestimate risk in low-risk patients and overestimate it in high-risk cases^18^. Recent research has focused on biomarkers—including imaging^19,20^Ki-67^21^, hematological markers (e.g., NLR), and urinary immune cells like T cells and MDSCs—for more personalized risk assessment^6,22–24^. In this study, we evaluated the prognostic value of NLR and LMR from blood counts, and urinary T cell, Neutrophil and MDSCs levels, noting that only one prior study addressed this specific urinary marker association in BCG-treated patients.

BCG therapy and second TURBT has been shown to reduce the recurrence of BC^25,26^. According to 2006 EORTC data, 1-year recurrence rates range from 15 to 61%^15^. In our 30-month follow-up, only 12 patients (15%) experienced recurrence. This lower rate compared to previous studies may be due to the absence of BCG therapy in the comparator study and the lack of routine second TURBT at that time.

 Nomogram studies for predicting recurrence and progression in NMIBC patients commonly highlight factors such as age, sex, tumor number and size, presence of concomitant CIS, and the stage and grade of the primary tumor. While these variables show varying significance across models, external validations often report recurrence and progression rates that differ from predictions^27^. In our analysis, these factors did not differ significantly between patients with and without recurrence. This may be due to the small sample size and the relatively short follow-up period, which limited the number of recurrence events.

 Clinical studies have identified NLR and LMR—markers of systemic inflammation—as independent predictors of recurrence and progression in various cancers, including NMIBC^28,29^. In our study, however, NLR and LMR did not differ significantly between patients with and without recurrence. This may be due to the small sample size, short follow-up period.

 TME is a complex system composed of cancer cells, stromal tissue (including blood vessels, immune cells, fibroblasts, and signaling molecules), and the extracellular matrix^30^. Interactions between cancer cells and the TME play a key role in tumor growth, invasion, resistance to therapy, and poor prognosis^31^. Neutrophils in the TME are considered pro-tumor factors, and higher peripheral neutrophil counts have also been linked to worse outcomes^32^. T cells, another important immune component, are detected in urine following BCG therapy, reflecting an active immune response^33^. In our study, we analyzed the urinary neutrophil-to-T cell ratio, as urine samples are thought to reflect the TME. This ratio was significantly higher in patients with recurrence, both before the first BCG instillation and around the sixth dose. While the ratio was also higher after the first dose in the recurrence group, the difference was not statistically significant. This analysis represents one of the most distinctive aspects of our study. These findings suggest that the local immune environment within the bladder—captured through urinary analysis—may reveal prognostically relevant immunological patterns that are not detectable in systemic (peripheral blood) markers.

 T cells are key immune components in the tumor microenvironment (TME), with subtypes such as cytotoxic (CD8+), helper (CD4+), and regulatory T cells (Tregs). Increased CD8 + T cell presence is generally linked to better prognosis, while Tregs are associated with immunosuppression. Other immunosuppressive elements in the TME include tumor-associated macrophages, neutrophils, and myeloid-derived suppressor cells (MDSCs)^30,34^. In our study, the T/MDSCs ratio in the non-recurrence group significantly increased after the sixth BCG instillation compared to baseline, suggesting a stronger anti-tumor immune response. Although a similar trend was observed in the recurrence group, it was not statistically significant, possibly indicating weaker immune activation. Additionally, the proportion of MDSCs decreased after BCG treatment in the non-recurrence group but increased in the recurrence group, implying a shift toward greater immunosuppression in patients who relapsed.

 Despite its strengths, our study has some limitations. The sample size was relatively small, and the short follow-up period may have missed later recurrences. We also did not analyze individual T cell subtypes, which can have varying effects on tumor progression, nor did we include other immunosuppressive cell populations in our flow cytometry analysis. We do not claim superiority over existing nomograms but propose our approach as an innovative, non-invasive alternative that may enhance recurrence prediction—especially when combined with conventional clinical markers. Future multicenter studies with longer follow-up and larger patient cohorts will be needed to validate these findings and better assess their long-term predictive value.

## Conclusİon

 In conclusion, our study suggests that changes in urinary immune cell ratios reflect the immunomodulatory effects of BCG therapy and may be associated with recurrence outcomes in NMIBC. A significant increase in the T/MDSCs ratio and a decrease in MDSCs/Total cell ratio were observed only in the non-recurrence group, indicating a more effective anti-tumor response. Although peripheral NLR was not predictive, the urinary neutrophil-to-T cell ratio was consistently higher in patients with recurrence and reached significance at multiple time points. These findings highlight the potential of urinary immune markers as early predictors of recurrence and support the need for validation in larger cohorts to guide individualized treatment strategies.

## Data Availability

The datasets used and/or analyzed during the current study are available from the corresponding author onreasonable request.
